# *Bartonella* DNA in heart tissues of bats in central and eastern Europe and a review of phylogenetic relations of bat-associated bartonellae

**DOI:** 10.1186/s13071-018-3070-7

**Published:** 2018-08-29

**Authors:** Alexandra Corduneanu, Attila D. Sándor, Angela Monica Ionică, Sándor Hornok, Natascha Leitner, Zoltán Bagó, Katharina Stefke, Hans-Peter Fuehrer, Andrei Daniel Mihalca

**Affiliations:** 1 Department of Parasitology and Parasitic Diseases, University of Agricultural Sciences and Veterinary Medicine of Cluj-Napoca, Cluj Napoca, Romania; 2 Department of Parasitology and Zoology, University of Veterinary Medicine, Budapest, Hungary; 3 Department of Pathobiology, Institute of Parasitology, University of Veterinary Medicine, Vienna, Austria; 4 Institute for Veterinary Disease Control, Austrian Agency for Health and Food Safety (AGES), Mödling, Austria; 5 Museum of Natural History, Vienna, Austria

**Keywords:** Bacterial pathogens, *Bartonella* spp., Diversity, Heart tissues, *Myotis*, *Pipistrellus*

## Abstract

**Background:**

Bats are among the most widely distributed mammals worldwide and can represent hosts or reservoirs for a number of different pathogens. *Bartonella* spp. are opportunistic bacterial pathogens, which are transmitted by a large variety of arthropods. The aim of this study was to investigate the presence and host-associations of these Gram-negative bacteria in heart tissues of bats collected in four different countries from eastern and central Europe and to analyze their phylogenetic relationship with other bat-associated bartonellae.

**Results:**

The results of this study show for the first time the presence of *Bartonella* spp*.* DNA in heart tissues of bats from central and eastern Europe. The overall prevalence of the infection was 1.38%. Phylogenetic analysis identified four new *Bartonella* spp. sequences, which were closely related with other *Bartonella* previously isolated from bats in Europe and North America.

**Conclusions:**

The *glt*A sequences of *Bartonella* spp. showed considerable heterogeneity in the phylogenetic analysis resulting in six different clades. Our study demonstrated the presence of *Bartonella* spp. only in heart tissues of bats from Romania, with two new bat species recorded as hosts (*Myotis* cf*. alcathoe* and *Pipistrellus pipistrellus*).

**Electronic supplementary material:**

The online version of this article (10.1186/s13071-018-3070-7) contains supplementary material, which is available to authorized users.

## Background

Bats are among the most widespread mammalian species worldwide with high local diversity and abundance. They are divided in two suborders: Yinpterochiroptera with distribution especially in the tropical regions and Yangochiroptera more widely distributed and with higher species diversity [1]. They are unique among mammals, as they have the ability to fly, even for long distances during the migration periods [2, 3]. Moreover, they can live in dense colonies, sometimes consisting in several bat species. Bats can adapt to various environmental conditions, and act as potentially important reservoir hosts for multiple pathogens, including zoonotic ones [4]. Multiple studies demonstrated their role as natural reservoirs for different pathogens including viruses [5–7], bacteria [8, 9] and parasites [10–12].

The genus *Bartonella* is a relatively diverse group of Gram-negative, facultative intracellular, haemotropic, vector-borne, bacteria that infect a wide-range of mammals and have a global distribution. After infection, the bacteria eventually enter the erythrocytes and endothelial cells and can persist asymptomatically in a wide range of mammalian reservoir hosts such as rodents, insectivores, carnivores, and ungulates [13–15]. The infection is transmitted mainly by arthropod vectors including fleas [16], sand flies [17], lice [18], mites [19] and ticks [20, 21]. The transmission and evolution of *Bartonella* species in mammals is the result of a complex relationship between multiple hosts, vectors and pathogens. There are many species of *Bartonella*, some of them with a large host spectrum and zoonotic potential (i.e. *B. henselae*, *B. grahamii*, *B. elizabethae*, *B. koehlerae* and* B. rochalimae*) while some others are known only from single host species [22–24].

*Bartonella* spp. has been reported with different prevalence and a high genetic diversity in bats and bat flies [25–29]. However, the knowledge on the occurrence of *Bartonella* in tissues of bats is still scarce. In Europe there are two studies reporting the presence of *Bartonella* spp. in bat tissues, involving different species [30, 31]. Both are geographically located at the margins of the continent (UK *vs* Georgia). Bai et al. [9] found 35 % of 218 bats positive for *Bartonella* DNA and more than 25 genetic variants were identified. Urushadze et al. [30] investigated the presence of *Bartonella* in the blood of 212 live bats by culture followed by PCR and found a 49.5 % prevalence.

Considering all these, the aim of our study was to demonstrate the presence and diversity of *Bartonella* spp. in heart tissues of different species of bats from central and eastern Europe. We primarily targeted bat species which are rarely recorded in caves (and are less represented in epidemiological studies), with accent on building-dwelling bats, the group with the highest contact rate with humans and potentially posing a zoonotic risk.

## Methods

A total of 435 carcasses were collected from different countries from central and eastern Europe (Austria, Czech Republic, Hungary and Romania) between 2001 and 2016 (Additional file 1: Table S1). The samples were collected from carcasses of bats accidentally killed (collision with man-made structures, road kills) or that had died of natural causes (e. g. hypothermia caused by early spring emergence) and stored in freezer at -20 °C (samples from Czech Republic, Hungary and Romania) or at -80 °C (samples from Austria) until their necropsy. From each bat the heart was collected, as this was the only tissue available from all animals. No live bat was harmed or killed for the purpose of this study. Bats were identified to species level using morphological keys [31]. Genomic DNA was extracted from 25 mg of heart tissue using DNeasy Blood & Tissue Kit (Qiagen, Hilden, Germany) according to the manufacturer’s instructions using 200 μl of elution buffer and stored at -20 °C.

A PCR targeting the 370 bp of the *glt*A encoding gene was employed, using the following primers: CSH1f (5'-GCG AAT GAA GCG TGC CTA AA-3') and BhCS.1137 (5'-AAT GCA AAA AGA ACA GTA AAC A-3') [32]. The reactions were carried out in 25 μl reaction mixture which contained 12.5 μl 2× Green Master Mix (Rovalab GmBH, Teltow, Germany), 6.5 μl water, 1 μl of each primer (0.01 mM final concentration) and 4 μl aliquot of isolated DNA. The PCR was performed using the T1000™ Thermal Cycler (Bio-Rad, Hercules, CA, USA) with the following conditions: initial denaturation at 95 °C for 5 min, followed by 35 cycles of denaturation at 95 °C for 30 s, annealing at 52.5 °C for 30 s and extension at 72 °C for 30 s and a final extension at 72 °C for 10 min. For each set of reactions (45 samples), 2 negative controls (PCR water) and one positive control which was DNA obtained from a *Bartonella henselae*, strain (ID 54A) isolated from a cat from Israel [33]. Amplification products were visualized by electrophoresis on 1.5% agarose gel stained with RedSafe™ 20,000× Nucleic Acid Staining Solution (Chembio, St Albans, UK), and their molecular weight was assessed by comparison to a molecular marker (Hyperladder IV, Bioline, London, UK). PCR products were purified using a commercial kit (Isolate II PCR and Gel Kit, Bioline, London, UK) and sent for sequencing with the primers described above in both directions (Macrogen Europe, Amsterdam, Netherlands).

The sequences were compared with those available in GenBank using Basic Local Alignments Tool (BLAST) analysis. The evolutionary history was inferred by Maximum Likelihood method based on the Tamura-Nei model [34]. The *glt*A gene has been shown to be suitable for phylogenetic analysis among *Bartonella* species [35] and is currently the most widely used to detect *Bartonella* infection. Using the search query keywords ‘*Bartonella* bats *glt*A’, we retrieved from GenBank all the sequences available from bats and their ectoparasites. Furthermore, based on the available literature concerning bartonellae from bats, we produced a database, where, from each unique *Bartonella glt*A genotype found, we included data on the host species and the species of the ectoparasite, in the case they were present (Additional file 2: Table S2). For phylogenetic analyses, as the lengths of the downloaded *glt*A sequences were different, they were trimmed to a length of 232 base pairs. In total, the phylogenetic analysis included 210 unique *Bartonella* genotypes from bat flies as well as from bats belonging to 8 families from both suborders. *Brucella melitensis* was chosen as outgroup, as it is also an Alphaproteobacteria from the order Rhizobiales.

Statistical analysis was performed using EpiInfo™ 7 (CDC, USA) software. The overall prevalence of *Bartonella* spp., the prevalence at locality level and the prevalence for each bat species and their 95% confidence interval (95% CI) were calculated.

## Results

Overall, 435 samples were tested for the presence of *Bartonella* spp. DNA. A total of 6 samples were positive (1.38%). The positive samples belonged to three bat species: *Myotis* cf. *alcathoe* (3/12; 25%), *Nyctalus noctula* (2/228; 0.88%) and *Pipistrellus pipistrellus* (1/68; 1.47%). The following species were negative (numbers of examined bats in parentheses): *Barbastella barbastellus* (*n* = 2); *Eptesicus nilssonii* (*n* = 1); *E. serotinus* (*n* = 6); *Hypsugo savii* (*n* = 9); *Miniopterus schreibersii* (*n* = 4); *My.bechsteinii* (*n* = 4); *My.* cf. *brandtii* (*n* = 3); *My. daubentonii* (*n* = 2); *My. myotis* (*n* = 6); *My.* cf. *mystacinus* (*n* = 4); *My. nattereri* (*n* = 1); *Nyctalus leisleri* (*n* = 5); *Pipistrellus kuhlii* (*n* = 8); *Pi. nathusii* (*n* = 28); *Pi. pygmaeus* (*n* = 5); *Plecotus auritus* (*n* = 7); *Pl. austriacus* (*n* = 1); *Rhinolophus euryale* (*n* = 9); *R. ferrumequinum* (*n* = 1); *R. hipposideros* (*n* = 1); and *Vespertilio murinus* (*n* = 20).

All positive samples (*n* = 6) originated from three locations in Romania: Muntele Puciosu (3/31; 9.68%), Cheile Bicazului (2/92; 2.17%) and Huda lui Papară (1/68; 1.47%) (Table 1).

**Table 1:** Distribution and location of sample tested

Country	Location	*n*	*Bartonella* spp.
Austria	Baden	1	–
	Hermagor	1	–
	Hollabrun	1	–
	Klosterneuburg	1	–
	Korneuburg	3	–
	Mauerbach	2	–
	Mödling	4	–
	Neulengbach	1	–
	Salzburg	1	–
	Stockerau	1	–
	Telfs Innsbruck Land	1	–
	Tulln	1	–
	Vienna	42	–
	Winer Neustadt	1	–
Czech Republic	Brno	39	–
	Heroltovice	1	–
	Malá Morávka	1	–
	Ochoz	3	–
	Znojmo	1	–
Hungary	Edelény	9	–
	Eger	19	–
Romania	Babadag	47	–
	Bucureşti	8	–
	Cheile Bicazului	88	Yes
	Huda lui Papară	68	Yes
	Iaşi	50	–
	Muntele Puciosu	30	Yes
	Peştera cu Apă din Valea Leşului	1	–
	Peştera Meziad	1	–
	Peştera Liliecilor- Bistriţa Monastery	1	–
	Sântu Gheorghe	1	–
	Sibiu	1	–
	Peştera Tăuşoarele	1	–
	Tulcea	1	–
	Ugron	1	–

*Abbreviation*: *n* number of samples collected

The analysis of the sequences showed that two from Muntele Puciosu and two from Cheile Bicazului were identical to each other, resulting in 4 unique sequences. The four sequences differed from each other by 6–24 nucleotides (Table 2).

**Table 2:** Differences regarding the number of nucleotides between sequences isolated in Romania

	MG914431.1	MG914432.1	MG914433.1
Distance			
MG914432.1	0.032	–	–
MG914433.1	0.009	0.038	–
MG914434.1	0.009	0.044	0.018
No. of nucleotides			
MG914432.1	20	–	–
MG914433.1	6	21	–
MG914434.1	6	24	11

BLAST analysis of the *glt*A sequences showed 96– 98% similarity to different sequences, isolated from bats in Europe (Georgia, GenBank: KX300154.1 and KX300200.1; and UK, GenBank: AJ871614.1) (Table 3). All sequences were submitted to the GenBank database under the accession numbers MG914431-MG914434.

**Table 3:** Results of the BLAST analysis

Sequence ID	Identity (%)	Acc. no.	Origin	Host
MG914431	98	KX300154.1	Georgia	*Myotis emarginatus*
MG914432	97	KX300200.1	Georgia	*Eptesicus serotinus*
MG914433	96	KX300154.1	Georgia	*Myotis emarginatus*
MG914434	96	AJ871614.1	UK	*Pipistrellus* sp.

**Fig. 1:**
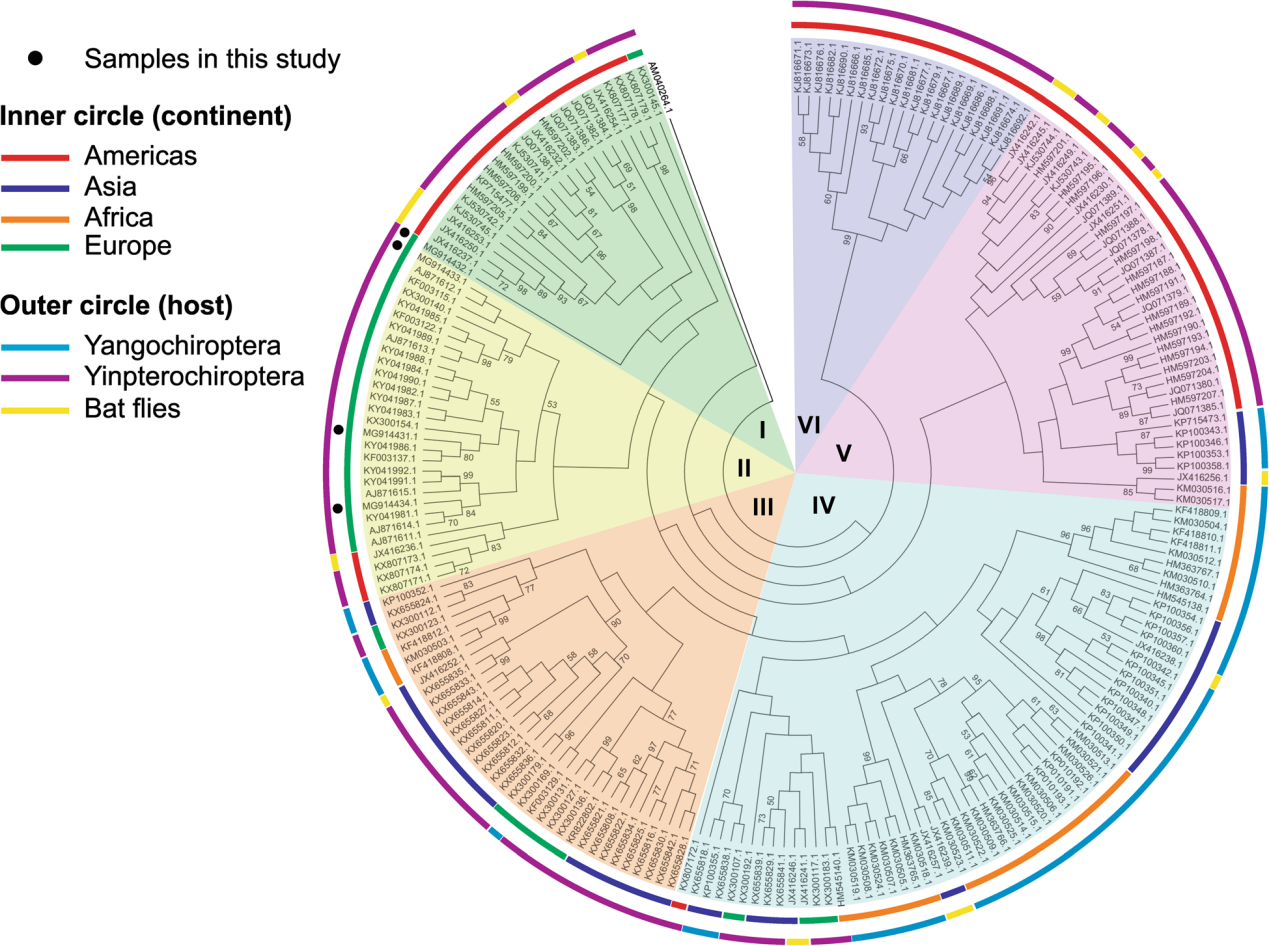
Phylogenetic tree of bat associated bartonellae. The evolutionary history was inferred by using the Maximum Likelihood method based on the Tamura-Nei model [34]. The bootstrap consensus tree inferred from 1000 replicates [56] is taken to represent the evolutionary history of the taxa analyzed [56]. Branches corresponding to partitions reproduced in less than 50% bootstrap replicates are collapsed. Initial tree(s) for the heuristic search were obtained automatically by applying Neighbor-Join and BioNJ algorithms to a matrix of pairwise distances estimated using the Maximum Composite Likelihood (MCL) approach, and then selecting the topology with superior log-likelihood value. The analysis involved 210 nucleotide sequences. There were a total of 232 positions in the final dataset. Evolutionary analyses were conducted in MEGA7 [57]

The global molecular phylogenetic analysis using the *glt*A sequences of *Bartonella* spp. isolated from bats in different parts of the world showed the presence of six major clades (Table 4, Fig. 1).

**Table 4:** Hosts and geographical distribution of the six major clades of bat-associated bartonellae

Clade	Host order	Host family	Geographical distribution
I	Yangochiroptera	Phyllostomidae	Central and South America
		Mormoopidae	Central and South America
		Vespertilionidae	North America, Europe
II	Yangochiroptera	Vespertilionidae	North America, Europe
III^a^	Yangochiroptera	Vespertilionidae	Asia, Europe
	Yinpterochiroptera	Pteropodidae	Africa
		Rhinolophidae	Africa, Asia
IV^a^	Yangochiroptera	Miniopteridae	Africa
		Vespertilionidae	Asia, Europe, North America
	Yinpterochiroptera	Hipposideridae	Africa, Asia
		Pteropodidae	Africa
		Rhinolophidae	Africa, Asia
V^a^	Yangochiroptera	Noctilionidae	South America
		Phyllostomidae	Central and South America
	Yinpterochiroptera	Pteropodidae	Africa
		Rhinolophidae	Asia
VI	Yangochiroptera	Phyllostomidae	Central America
		Vespertilionidae	Central America

^a^Present also sequences from bat flies

The first clade consisted in *Bartonella* spp. genotypes isolated from bats or bat flies in the Americas as well as sequences of the zoonotic pathogen *B. mayotimonensis* but also one of the sequences isolated from a bat in Romania. The other three sequences of *Bartonella* spp. in our study clustered in the second clade, together with various sequences isolated from Europe (Finland, France, Georgia, Spain and the UK) and four sequences isolated from North America. The third cluster consisted in different sequences isolated from the Old World (Asia, Europe and Africa). The fourth clade was the largest and most diverse and included sequences isolated from four different continents. The fifth clade comprised sequences from both Old World and New World, while the sixth clade consisted exclusively in sequences from South America, belonging to Yangochiroptera (Fig. 1).

## Discussion

This study investigated the presence, prevalence and genetic diversity of *Bartonella* spp. in insectivorous bats from three different countries from central and eastern Europe and is the first evidence of the presence of these bacteria in heart tissues of bats from eastern and central Europe. This is the first study where *My.* cf. *alcathoe* and *Pi. pipistrellus* were found positive for *Bartonella* spp., while *Ny. noctula* was previously reported to harbour this group of pathogens [36, 37]. Multiple bat species may share the same *Bartonella* species without evident host specificity [38, 39] or they can harbour one or few *Bartonella* species-specific for a particular bat species [25, 36, 40, 41].

Reports of *Bartonella* infections are known from blood of bats from various countries across the world with different prevalence. High prevalence was reported in Georgia [30], Taiwan [42], Guatemala [38], Costa Rica [27], Kenya [40] and China [43], compared with a low prevalence in South Africa, Swaziland [29] and the USA [44]. Most of the studies were focused on the detection of *Bartonella* spp. in blood and bat associated ectoparasites [45, 46], but researchers from Argentina [47], France, Spain [37], Georgia [9] and the UK [36] tested also tissues for the presence of the bacteria. The positive bats from France, Spain and the UK, together with our positive samples belonged to the family Vespertilionidae, which contain high number of building-dwelling bats species. The positive bats from Argentina and Georgia belonged to three different bat families, the Molossidae, Rhinolophidae and Vespertilionidae, with all the analysed bats were cave-dwelling species. On the family level, the prevalence of *Bartonella* was estimated to be between 7.3% on species of the family Nycteridae and 54.4% on species of the Miniopteridae [37]. The report of low prevalence of *Bartonella* DNA in bats from Romania may be the result that we targeted only one molecular marker (the *glt*A gene) instead of multiple markers [48] and the majority of bat species analyzed are rarely parasitized by bat flies, which are suggested to be the main vectors for *Bartonella* sp. [49].

The global phylogenetic analysis of the sequences considered in this study showed that there is a high diversity among *Bartonella* isolated from bats and their ectoparasites. The distribution of *Bartonella* spp. in different bat families depends also on the geographical distribution of that particular family (Table 4). Three of the clades (I, II and VI) include only *Bartonella* spp. isolated from Yangochiroptera. The most diverse clade regarding the number of bat host families was clade IV: the Miniopteridae and Vespertilionidae (Yangochiroptera) and the Hipposideridae, Pteropodidae and Rhinolophidae (Yinpterochiroptera) (Table 4). Sequences of *Bartonella* spp. isolated from bats belonging to the family Vespertilionidae were present in five out of six clades (all except clade V), as this family is among the most diverse, widespread and well-studied. In Europe there are 44 bat species out of which 35 belong to Vespertilionidae [31] and all the studies conducted in this part of the Old World for detection of *Bartonella* spp. were focused mainly on this family [36, 37, 50, 51]. Our study was performed on various bat species, with the positive samples belonging to the family Vespertilionidae and the negative belonging to the families Miniopteridae and Rhinolophidae.

So far, the pathogenicity of bat-associated bartonellae to humans remains unknown, and further studies are needed to clarify their zoonotic potential. There are reports from Finland and the USA where different Vespertilionidae bats harboured the human pathogen *B. mayotimonensis* [44, 50], which was originally detected in the resected aortic valve of a 59-year-old patient from the USA [41]. Stuckey et al. [37] suggested that studies regarding the detection of *Bartonella* spp. in bats should be focused especially on those belonging to the Vespertilionidae (genera *Nyctalus*, *Pipistrellus* and *Myotis*), as the *Bartonella* isolated from these genera seem to be genetically related to *B. mayotimonensis*. Although all the positive samples from Romania were isolated from species of the family Vespertilionidae, our study did not reveal sequences related with any of the zoonotic *Bartonella* genotypes.

Diverse genetic variants of *Bartonella* were found in bats and their associated bat flies, suggesting that the latter may act as vectors. *Bartonella* spp. prevalence is higher in bat ectoparasites and have a much more genetic diversity compared with those isolated from the bats [26, 28, 38, 39, 42, 49, 50, 52–55].

## Conclusions

This study showed that bats can harbour different strains of *Bartonella* spp., but with a low prevalence, reporting the presence of these bacteria in two new hosts (*My.* cf*. alcathoe* and *Pi. pipistrellus*). The molecular phylogenetic analysis conducted in this study revealed a high genetic diversity among *Bartonella* spp. isolated from bats in different parts of the world, with the presence of six major clades.

## Additional files

Additional file 1: **Table S1.** Samples distribution according to locality and species. (XLSX 14 kb)

Additional file 2: **Table S2.** Detailed information regarding the sequences used in the phylogenetic analysis. (XLSX 29 kb)

## Abbreviations

BLAST: Basic Local Alignment Search Tool
